# Liquid–Liquid Phase Separation in the Presence of Macromolecular Crowding and State-dependent Kinetics

**DOI:** 10.3390/ijms22136675

**Published:** 2021-06-22

**Authors:** Alick-O. Vweza, Chul-Gyu Song, Kil-To Chong

**Affiliations:** 1 Department of Electronics and Information Engineering, Jeonbuk National University, Jeonju 54896, Korea; alick@jbnu.ac.kr (A.-O.V.); cgsong@jbnu.ac.kr (C.-G.S.); 2 Advanced Biomedical Imaging Center, Jeonbuk National University, Jeonju 54896, Korea; 3 Advanced Electronics and Information Research Center, Jeonbuk National University, Jeonju 54896, Korea

**Keywords:** biomolecular condensates, macromolecular crowding, membraneless organelles, liquid–liquid phase separation, intrinsically disordered proteins, state-dependent reactions

## Abstract

Biomolecular condensates formed via liquid–liquid phase separation (LLPS) are increasingly being shown to play major roles in cellular self-organization dynamics in health and disease. It is well established that macromolecular crowding has a profound impact on protein interactions, particularly those that lead to LLPS. Although synthetic crowding agents are used during in vitro LLPS experiments, they are considerably different from the highly crowded nucleo-/cytoplasm and the effects of in vivo crowding remain poorly understood. In this work, we applied computational modeling to investigate the effects of macromolecular crowding on LLPS. To include biologically relevant LLPS dynamics, we extended the conventional Cahn–Hilliard model for phase separation by coupling it to experimentally derived macromolecular crowding dynamics and state-dependent reaction kinetics. Through extensive field-theoretic computer simulations, we show that the inclusion of macromolecular crowding results in late-stage coarsening and the stabilization of relatively smaller condensates. At a high crowding concentration, there is an accelerated growth and late-stage arrest of droplet formation, effectively resulting in anomalous labyrinthine morphologies akin to protein gelation observed in experiments. These results not only elucidate the crowder effects observed in experiments, but also highlight the importance of including state-dependent kinetics in LLPS models, and may help in designing further experiments to probe the intricate roles played by LLPS in self-organization dynamics of cells.

## 1. Introduction

Biologically regulated liquid–liquid phase separation (LLPS) is emerging as one of the major processes that facilitates the mixing and demixing of intracellular space into two or more distinct phases to form molecular assemblies termed biomolecular condensates [1,2,3,4,5,6,7,8,9,10,11,12,13]. The liquid-like features of the resulting membraneless organelles (MLOs), such as nucleoli and stress granules, enable these condensates to form coherent structures that sub-compartmentalize and concentrate a specific set of biomolecules, thereby spatially organizing biochemical reactions [14,15,16]. LLPS has been shown to be the main process that drives the formation of heterochromatin, a repressive DNA domain [17,18] that plays major roles in nuclear architecture, DNA repair and genome stability [19]. In epithelial and endothelial cell layers, zonula occludens proteins have been shown to drive the formation of tight junctions through self-organization via LLPS [20,21]. In vitro experiments have also linked the LLPS of Atg proteins to mediate the organization of autophagy machinery in yeast [22,23]. Recent studies have extended biomolecular condensates to include the postsynaptic density (PSD) in neuronal synapses that regulate synaptic signaling and plasticity [24,25,26,27]. In addition to playing major roles in normal cellular function, the emergent properties from LLPS processes have been implicated in the onset of an array of pathophysiological conditions such as neurodegeneration [11,28,29,30,31,32,33,34,35,36,37] and carcinogenesis [38,39] due to the aberrant phase separation that triggers protein gelation and aggregation in the condensed phases.

In vitro studies on LLPS have shown that the phase-separated condensates form highly spherical droplets, coalesce upon contact with each other, and show a rapid exchange of molecular materials among themselves and the surrounding bulk material [3,7,9,40]. The dynamics governing these liquid-like features are akin to classical first-order phase transitions. Generally, the LLPS of proteins is strongly linked to the concentration and type of macromolecules and the environmental conditions such as temperature, ionic strength, pH and macromolecular crowding [41]. In particular, macromolecular crowding has been shown to lower diffusion and alter forward and reverse enzymatic reactions in the cytoplasm [42,43,44]. Using synthetic macromolecular crowding agents such as polyethylene glycol (PEG), Ficoll and dextran [45], crowding has been shown to induce LLPS, co-condense phase-separated biomolecules, and alter the biophysical properties of condensed phases [46,47,48]. However, the challenge of using these labeled tracer agents to characterize the intracellular dynamic is the fact that these tracers may not capture the composition and behavior of unlabeled macromolecules that affect the labeled molecules within the vicinity [45]. Effectively, these observations point to the importance of further work to understand the effects of macromolecular crowding in regulating cellular physiology in general and LLPS in particular.

One way to further understand the roles crowders play in LLPS is by augmenting experimental and theoretical studies. Several theoretical models have been applied to explore LLPS and the properties of resulting MLOs [9,14,49,50,51]. These models can be broadly grouped into either field-theoretic or particle-based categories. A description of these categories can be found in the review by Falahati et al. [15]. Of particular relevance to this work are the models of Berry et al. [9], Gasior et al. [50], Shin et al. [52] and IIker et al. [51], where the authors apply the Cahn–Hilliard or its modified version and Flory–Higgins theory to study LLPS in binary and ternary systems. Although ideal chemical kinetics are used in some of these models, none include the effects of macromolecular crowding on phase separation dynamics. Additionally, simple constant-rate chemical kinetics used are physically constrained in capturing the nonequilibrium nature of complex biochemical reactions that drive intracellular phase separation [53]. In particular, macromolecular crowders may bias the reactions either towards products or reactants depending on changes in the free energy of the reaction [54]. Furthermore, the presence of macromolecular crowders has been shown to either speed up, cause no change to, or slow down LLPS when compared to crowder-free solutions [55].

In order to overcome some of the aforementioned challenges, we herein extend the conventional Cahn–Hilliard model for phase separation in binary mixtures by coupling it to experimentally derived macromolecular crowding dynamics [56] and state-dependent reaction kinetics [57]. Through extensive field-theoretic computer simulations, we show that the inclusion of macromolecular crowding results in the late-stage coarsening and stabilization of relatively smaller droplets. Additionally, at high crowder concentration, the model predicts an accelerated growth and coarsening of droplets. However, any further increase in crowder concentration results in the reactions arresting the late-stage coarsening of droplets, thereby effectively forming anomalous labyrinthine morphologies akin to gel-like protein aggregates observed in the experiments. These results not only elucidate crowder effects observed in experiments but also highlight the importance of including state-dependent kinetics in LLPS models, and may help in designing further experiments to probe the intricate roles played by LLPS in the self-organization dynamics of cells.

## 2. Methods

### 2.1. Phase Separation Dynamics

**Figure 1:**
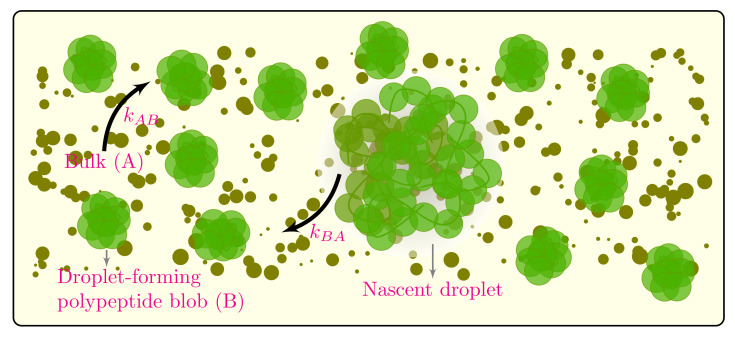
A cartoonistic representation of phase separation and bulk–polypeptide interaction kinetics in a macromolecular crowded environment under poor solvent regime. The polypeptides are assumed to be under local thermal equilibrium such that blob–solvent interactions result in the proteins taking globular conformations. At critical concentrations of the macromolecules, the polypeptide blobs go through a phase separation process whereby blobs aggregate into droplets with the blobs leaving the bulk at a rate $k_{AB}$ and disassociating from the droplet at a rate $k_{BA}$.

We consider the intracellular space as a metastable poor solvent continuum fluid made up of droplet-forming polypeptide blobs (component *B*) and all other molecules making up the cyto- or nucleoplasm, the bulk (component *A*), as shown in Figure 1. The polypeptides are assumed to be under local thermal equilibrium such that blob–solvent interactions result in the proteins taking globular conformations. At critical concentrations of the macromolecules, the polypeptide blobs go through a phase separation process whereby blobs aggregate into droplets with the blobs leaving the bulk at a rate $k_{AB}$ and disassociating from the droplet at a rate of $k_{BA}$. We define the spatiotemporal protein volume fraction (order parameter) at position r and time *t* as $\phi \left(r,t\right)=\phi_{B}\left(r,t\right)/\left(\phi_{A}\left(r,t\right)+\phi_{B}\left(r,t\right)\right)$, where $\phi_{A}\left(r,t\right)$ and $\phi_{B}\left(r,t\right)$ are, respectively, the volume fractions of bulk molecules and droplet-forming polypeptide blobs. We assume that the fluid is incompressible such that $\phi_{A}\left(r,t\right)+\phi_{B}\left(r,t\right)=1$. In the following analysis, we present the phase separation dynamics of the field variable $\phi \left(r,t\right)$, taking into consideration biologically relevant parameters such as the effects of reaction rates $k_{AB}$ and $k_{BA}$ and experimentally verified macromolecular crowding dynamics.

To take into consideration the non-equilibrium nature of the intracellular space, we employ the modified Cahn–Hilliard model for the phase separation of a binary mixture by coupling the protein volume fraction to non-equilibrium state-dependent chemical kinetics according to [57]:

(1) $$\frac{\partial \phi \left(r,t\right)}{\partial t}=\nabla \cdot \left[M\left(\phi \right)\nabla \left(\frac{\delta F\left[\phi \right]}{\delta \phi }\right)\right]\mp R\left[\mu \left(\phi \right)\right],$$

where $M\left(\phi \right)$ is the volume fraction-dependent mobility, $F\left[\phi \right]$ is the free energy functional of the mixture, and $R\left[\mu \left(\phi \right)\right]$ is the reaction rate that dependents on the local chemical potential $\mu \left(\phi \right)=\partial F/\partial \phi$. We assume F(ϕ) to be a Flory–Huggins regular solution energy functional:

(2) $$F\left[\phi \right]=\int dr\left(f\left(\phi \right)+\frac{\kappa }{2}{\left|\nabla \phi \right|}^{2}\right),$$

where κ>0 is a parameter that relates the surface tension between A-rich and B-rich domains, and $f\left(\phi \right)$ is a double-well potential given by

(3) $$f\left(\phi \right)=\phi \ln\left(\phi \right)+\left(1-\phi \right)\ln\left(1-\phi \right)+\chi \phi \left(1-\phi \right).$$

In (3), the first two terms account for translational entropy due to the mixing between *A* and *B* species. The last term describes the interaction energy between molecules of *A* and *B* and is controlled by the enthalpic interaction parameter $\chi =\frac{z}{k_{B}T}\left(2\epsilon_{AB}-\left(\epsilon_{AA}+\epsilon_{BB}\right)\right)$. In mean-field description, $\epsilon_{AA}$, $\epsilon_{AB}$ and $\epsilon_{BB}$ are the nearest neighbor (effective coordinate number *z*) lattice interaction energies between monomers of species *A* and *B*. In the following analysis, we justify the choice of the concentration-dependent mobility M(ϕ) and the reaction rate $R\left[\mu \left(\phi \right)\right]$.

### 2.2. Macromolecular Crowding and Concentration-Dependent Mobility

**Figure 2:**
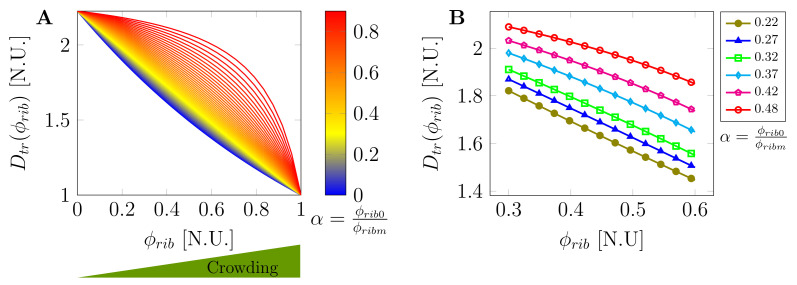
Dependence of tracer diffusion coefficient ($D_{tr}$) on macromolecular crowder concentration $\phi_{rib}$: (**A**) effective concentration-dependent tracer diffusion coefficient for several values of $\alpha \in \left[0,1\right]$; (**B**) dependency of $D_{tr}$ on crowder concentration for selected values of ribosome crowder concentration and control parameter in the range of α=0.35±0.13 in human cells (HEK293) as experimentally reported in [56].

Macromolecular crowding, the excluded volume effect of solutes in a solution [58], has been shown to severely reduce diffusion in the cytoplasm and affect both LLPS and the biophysical properties of formed biomolecular condensates [43,59,60]. More recently, ribosome concentration has been shown to be a major player in controlling cytoplasmic crowding by modulating the effective diffusion of biomolecules via the rapamycin complex 1 (mTORC1) pathway [56,61]. By applying osmotic stress experiments in human (HEK293) and *S. cerevisiae* cells, Delarue, et al. [56] introduced an experimentally verified physical model that predicts a cytoplasmic diffusion coefficient as a function of ribosome concentration, $\phi_{rib}$. The diffusion coefficient is modeled using the modified Doolittle Equation [62]:

(4) $$D\left(\phi_{rib}\right)=D_{0}\exp\left(-\zeta \frac{\phi_{rib}}{\phi_{ribm}-\phi_{rib}}\right),$$

where $D_{0}$ is the coefficient of diffusion in an infinitely diluted solution, $\phi_{ribm}$ is the maximum concentration of the crowding agents, and ζ is a scaling constant that is related to the strength of interaction of the tracer particle with its surrounding intracellular environment. Equation (4) is then modified by including normalized cell volume change due to macromolecular crowding to obtain the experimentally verified effective tracer diffusion coefficient as a function of crowder concentration (ribosomes):

(5) $$D_{tr}\left(\phi_{rib}\right)=\exp\left[\zeta \left(\frac{\alpha }{1-\alpha }\right)\left(\frac{1-\phi_{rib}}{1-\alpha \phi_{rib}}\right)\right],$$

where $\alpha =\phi_{rib0}/\phi_{ribm}$ and $\phi_{rib0}$ is the initial concentration of crowding macromolecules for a given volume of the intracellular space. In the case of ribosomes as the main crowding agents, the value of α has been shown to be in the range of 0.35±0.13, ζ=0.6±0.12 for human cells (HEK293) and 0.48±0.04, ζ=0.6±0.16 for *S. cerevisiae* cells [56]. Figure 2A shows the plot of $D_{tr}$ as a function of crowder concentration $\phi_{rib}$ for several values of $\alpha \in \left[0,1\right]$. As can be noted, the tracer diffusion coefficient effectively decreases with increasing volume fraction and this increase is relatively enhanced by macromolecular crowding agent. This can be seen in Figure 2B where the tracer diffusion coefficient has been plotted against the crowder concentration for the experimental range of α=0.35±0.13 for ribosome crowding agents in human cells (HEK293).

We employ model (5) to account for the effects of macromolecular crowding on LLPS and the properties of resulting morphologies. However, it is worth noting that the effective tracer diffusion coefficient $D_{tr}\left(\phi_{rib}\right)$ in (5) only takes into account the spontaneous diffusion of labeled tracer molecules (genetically encoded multimeric nanoparticles (GEMs) in this case [56]) and does not consider the effects of concentration gradients. However, the mobility $M\left(\phi \right)$ in (1) takes into account both the tracer diffusion and diffusion due to concentration gradients. The two are related by [63]

(6) $$M\left(\phi \right)=D_{tr}\left(\phi_{rib}\right)k_{B}T{\left(\frac{\partial^{2}f\left(\phi \right)}{\partial \phi^{2}}\right)}^{-1},$$

where $f\left(\phi \right)$ is the local potential defined in (3). Differentiating (3) twice and following the Vrentas formalism [64], we obtain:

(7) $$\begin{array}{cc} M\left(\phi \right) & =D_{tr}\left(\phi_{rib}\right)\frac{\phi \left(1-\phi \right)}{{\left(1-\phi \right)}^{2}{\left(\frac{D_{tr}\left(\phi_{rib}\right)}{ND\left(\phi \right)}\right)}_{\phi =0}+\phi \left(3-2\phi \right)}\\ \; & =D_{tr}\left(\phi_{rib}\right)\frac{\phi \left(1-\phi \right)}{\beta {\left(1-\phi \right)}^{2}+\phi \left(3-2\phi \right)}, \end{array}$$

where $\beta =\frac{1}{ND_{0}}$ and *N* is the degree of polymerization of phase-separating macromolecules. The last line is obtained by setting $\phi =\phi_{rib0}=0$ in (4) and (5).

**Figure 3:**
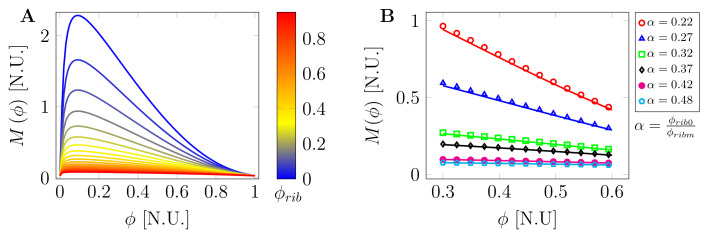
Mobility dependence on volume fraction for various crowder concentrations: (**A**) mobility against volume fraction at low and high ribosome crowder concentration, $\phi_{rib}$. At very high crowder concentration, the mobility is very low and almost constant for changes in volume fraction. The mobility sharply increases for a small change in volume fraction, particularly at low crowder concentration; and (**B**) changes in mobility at low crowder concentration $\phi_{rib}=0.2$ (solid lines) and high concentration $\phi_{rib}=0.8$ (marks) for various control parameters α in the experimental range from 0.22 to 0.48.

Figure 3 shows the plot of the mobility coefficient against the volume fraction for several values of macromolecular crowder concentration. As can be noted in Figure 3A, at very high crowder concentration ($\phi_{rib}\approx 1$), the mobility is very low and almost constant for changes in volume fraction of phase separating molecules. The mobility sharply increases for a small change in volume fraction, particularly at a low crowder concentration. Figure 3B shows changes in mobility at low crowder concentration $\phi_{rib}=0.2$ (solid lines) and high concentration $\phi_{rib}=0.8$ (marks) for various control parameters α in the experimental range from 0.22 to 0.48. As can be seen, this range of control parameter (α) has little effect on the mobility. This can be attributed to the observation that α=0.22:0.48 is far from the grass transition value of the intracellular space where α would be ∼1 [56]. In other words, under normal physiological conditions, the cyto-/nucleoplasm is far from glass transition.

### 2.3. State-Dependent Reaction Rates

Traditionally, biomolecular reaction rates are modeled using the celebrated law of mass action which is based on the assumption that the reaction rate is a state-independent constant [65]. In this paradigm, the interconversion between bulk species *A* and droplet forming blobs *B* (Figure 1) can be represented by the chemical reactions $A\underset{k_{AB}}{\overset{k_{BA}}{⇌}}B$. The law of mass action gives the rate of change of concentration as

(8) $$\frac{\partial \phi \left(r,t\right)}{\partial t}=k_{AB}\phi +k_{BA}\left(1-\phi \right),$$

where $k_{AB}$ and $k_{BA}$ are state-independent (ideal) reaction constants. However, in phase-separating systems such as polymeric blends, it has been shown that although these ideal chemical reactions tend to alter the spinodal decomposition [66,67], the reactions themselves introduce thermodynamic inconsistency in phase separation models [68,69]. Additionally, such state-independent reaction rates are physically constrained in capturing the nonequilibrium nature of complex biochemical reactions that drive intracellular phase separation. In particular, macromolecular crowders may bias the reaction rates either towards products or reactants depending on changes in the free energy of the reaction [54]. Therefore, in order to capture biologically relevant dynamics observed in vitro, the reaction rates $k_{AB}$ and $k_{BA}$ must incorporate the nonlinearities of thermodynamic driving forces that are responsible for phase separation. To this end, we apply thermodynamically consistent state-dependent chemical reactions first introduced for modeling phase transformations in inhomogeneous electrochemical systems [57]. The reaction rates are derived in terms of local chemical potential such that:

(9) $$R\left[\mu \left(\phi \right)\right]=k_{0}e^{-\mu^{\mathrm{ex}}/k_{B}T}\left[\exp\left(\sum_{r}\frac{s_{r}}{k_{B}T}\frac{\delta f\left(\phi \right)}{\delta \phi_{r}}\right)-\exp\left(\sum_{p}\frac{s_{p}}{k_{B}T}\frac{\delta f\left(\phi \right)}{\delta \phi_{p}}\right)\right],$$

where $\mu^{\mathrm{ex}}$ is the excess reactant-to-product activation barrier, $k_{0}$ is the rate constant, $s_{r}$ and $s_{p}$ are, respectively, the reactants and product stoichiometry coefficients. Note that if the thermochemical driving force $\left|\Delta \mu \left(\phi \right)\right|=\left|\sum_{p}\frac{s_{p}}{k_{B}T}\frac{\delta f\left(\phi \right)}{\delta \phi_{p}}-\sum_{r}\frac{s_{r}}{k_{B}T}\frac{\delta f\left(\phi \right)}{\delta \phi_{r}}\right|\ll k_{B}T$, then (9) can be expressed as

(10) $$R\left[\mu \left(\phi \right)\right]=k^{\prime }\Delta \mu \left(\phi \right),$$

where $k^{\prime }=\frac{k_{0}e^{-\mu^{\mathrm{ex}}/k_{B}T}}{k_{B}T}$. State-dependent reaction rates of this form have been shown to stabilize phase-separated protein clusters above the solubility curve, effectively pointing to the coexistence of more stable protein condensates of varying cluster sizes and composition [53].

Noting that $\phi_{r}=\phi_{A}=\phi$ and $\phi_{p}=\phi_{B}=1-\phi$ and following Bazant [57], the equilibrium condition $\delta f\left(\phi \right)/\delta \phi_{A}=\delta f\left(\phi \right)/\delta \phi_{B}$ for the regular solution free energy functional (3) yields $\phi /\left(1-\phi \right)=\exp\left(\chi \left(2\phi -1\right)\right)$. Combining this result with (9), we obtain the concentration-dependent dissipative reaction rate:

(11) $$R\left(\phi \right)=k_{0}\left[-\phi \exp\left(-\frac{\chi }{2}\left(2\phi -1\right)\right)+\left(1-\phi \right)\exp\left(\frac{\chi }{2}\left(2\phi -1\right)\right)\right].$$

Equation (11) shows that both the reaction and diffusion are driven by the same free energy functional (3) which decreases as the species phase separates according to the dynamics governed by (1).

**Figure 4:**
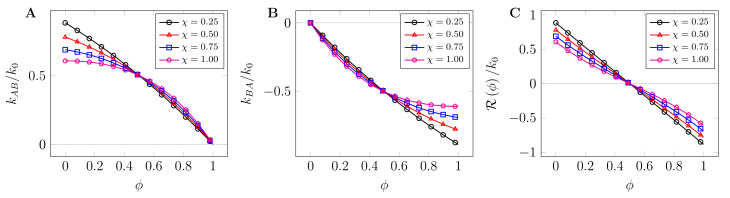
Relative dissipative state-dependent reaction rates plotted against volume fraction: (**A**) the relative forward reaction rate $k_{AB}/k_{0}$ decreases with increasing volume fraction due to decreased mobility; (**B**) similar observation is true for $k_{BA}/k_{0}$ which increases with increasing crowder volume fraction; and (**C**) overall contribution of the reaction rates, $R\left(\phi \right)$, which effectively reduces to zero at the equilibrium condition of the chemical potential. See the text for more details.

Figure 4 shows plots for relative reaction rates $k_{AB}/k_{0}$ (**A**) and $k_{BA}/k_{0}$ (**B**) as a function of volume fraction for several values of the interaction parameter χ. As can be noted, $k_{AB}/k_{0}$ decreases with increasing volume fraction due to decreased mobility. Intriguingly, the degeneracy of the reverse reaction rate $k_{BA}/k_{0}$ levels off at high volume fractions. This can be attributed to the observation that at low bulk protein concentrations, droplet-forming polypeptide blobs may easily diffuse out of the nascent droplet, thereby effectively disassembling it. In other words, due to the competing nature of the reactions, the rate $k_{BA}$ should quickly level off at high volume fraction and interaction rate. At a physicochemical level, this agrees with the observation that state-dependent reactions reduce chemical interconversion (dimers to monomers) inside a protein cluster, despite the dimer density being much higher inside the cluster than in the bulk [53]. When the chemical potential difference between the interconverting chemicals reduces to zero, the overall reaction rate $R\left(\phi \right)$ is zero, thereby effectively forming stable droplets, Figure 4C.

## 3. Computational Results and Discussion

The set of Equations (1), (3), (6) and (11) gives a minimal model of LLPS in a chemically reactive out-of-equilibrium intracellular space. In order to carry out the simulations, the free energy functional (2) is recast in the form:

(12) $$F\left[\phi \right]\leftarrow F\left[\phi \right]+\frac{R\left(\phi \right)}{2k_{0}}\int d^{2}r+\int d^{2}r^{\prime }\left(\phi \left(r,t\right)-\phi_{0}\right)G\left(r-r^{\prime },t\right)\left(\phi \left(r^{\prime },t\right)-\phi_{0}\right),$$

where $G\left(r-r^{\prime },t\right)$ is the Green’s function such that $\nabla^{2}G\left(r-r^{\prime },t\right)=-\delta \left(r-r^{\prime },t\right)$ [70] and the integration is over the simulation domain with appropriate boundary conditions to be discussed below. Note that written in the form (12), the dynamics (1) captures both long- and short-range interactions of the phase separating species. This is particularly important for biomolecular condensates where long-range interactions are inherent in intrinsically disordered proteins (IDPs) that go through LLPS [41,71,72]. With the help of (12), the dynamics (1) become:

(13) $$\frac{\partial \phi \left(r,t\right)}{\partial t}=\nabla \cdot \left[M\left(\phi \right)\nabla \left(\frac{\delta F\left[\phi \right]}{\delta \phi }\right)\right],$$

and can be written in dimensionless form by introducing r←r/L and $\tau \leftarrow \left(M_{0}/L^{2}\right)\tau$ where τ is a dimensionless time, *L* is the length scaling constant and $M_{0}$ is the diffusivity scaling constant. The simulation is performed in MATLAB™ on a 128×128 square grid using finite difference schemes for both temporal and spatial discretizations with periodic boundary conditions. The initial volume fraction used is $\phi \left(r,t\right)=\phi_{0}+\delta \phi \left(r,t\right)$ where $\phi_{0}$ is the initial average concentration and $\delta \phi \left(r,t\right)$ is a randomly generated infinitesimal concentration.

### 3.1. Evolution of Phase-Separated Morphologies

**Figure 5:**
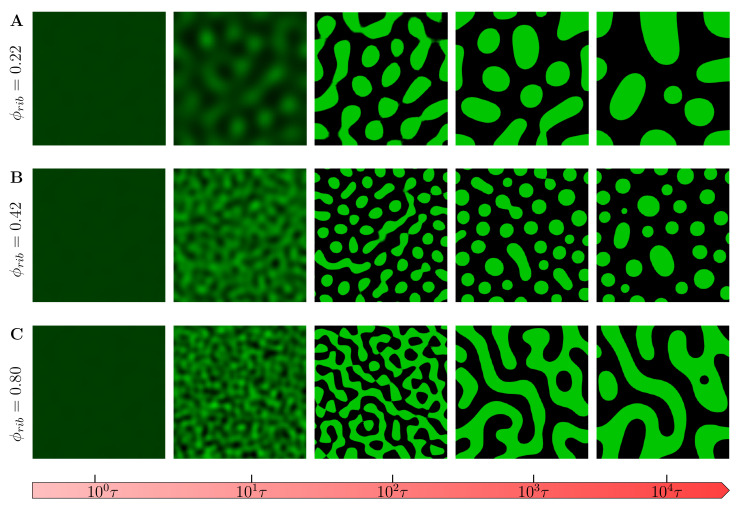
Evolution of the phase-separated morphology of the field variable $\phi \left(r,t\right)$ with the chemical reaction rate R(ϕ)=0 for selected crowder concentrations. (**A**) At very low crowder concentrations $\phi_{rib}=0.22$, the late-stage ($10^{4}\tau$) coarsened morphology is on average round droplets, as usually observed in in vitro experiments; (**B**) an increase in crowder concentration $\phi_{rib}=0.42$ results in smaller droplets being coarsened at late stage of the evolution of the field $\phi \left(r,t\right)$; and (**C**) any further increase in the crowder concentration results in the morphologies taking labyrinthine patterns with a smaller characteristic wavelength reminiscent of arrested gel-like phases (see Supplementary Materials Videos S1 and S2). Refer also to [73] where similar observations have been recently experimentally verified in peptide/oligonucleotide complexes.

We first studied the evolution of phase-separated morphology without chemical reactions. Figure 5 shows the evolution of the field variable $\phi \left(r,t\right)$ with the chemical reaction rate set to zero (R(ϕ)=0) from the same initial conditions ($\phi_{0}=0.01$) under different crowder concentrations. We observe that at very low crowder concentrations $\phi_{rib}=0.22$ (Figure 5A), the late stage ($t=10^{4}\tau$) coarsened morphologies are on average round droplets as is usually observed in in vitro experiments. However, an increase in crowder concentration $\phi_{rib}=0.42$ results in smaller droplets coarsened at late stage (Figure 5B) (see Supplementary Materials Videos S1 and S2). This can generally be attributed to the reduced mobility of droplet-forming polypeptides diffused in the overcrowded environment. This observation has also been more recently experimentally verified in peptide/oligonucleotide complexes [73]. Any further increase in the crowder concentration results in the phase-separated morphologies taking labyrinthine patterns with a smaller characteristic wavelength reminiscent of arrested gel-like phases (Figure 5C).

**Figure 6:**
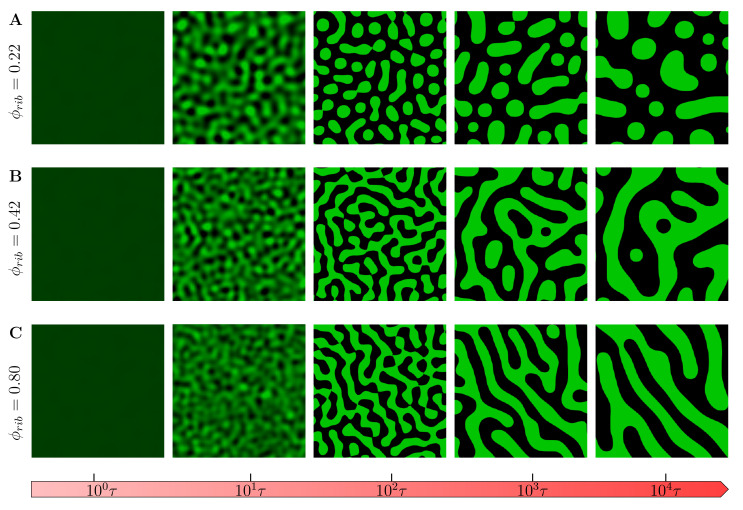
Evolution of phase-separated morphologies during LLPS with R(ϕ)≠0 for selected crowder concentrations. At a very low crowder concentration (**A**), the coarsened final regimes assume droplet-like patterns as in the case with no chemical reactions. However, the morphology quickly transitions to labyrinthine patterns with a much smaller late-stage characteristic length-scale (**B**,**C**). See also Supplementary Materials Videos S3 and S4.

The inclusion of chemical reactions results in phase-separated morphologies, as shown in Figure 6. At a very low crowder concentration (Figure 6A), the coarsened final regimes assume droplet-like patterns as in the case with no chemical reactions. However, the morphology quickly transitions to labyrinthine patterns with a much smaller late-stage characteristic length-scale. This is also further elaborated in the following paragraphs. Please also refer to Supplementary Materials Videos S3 and S4. It is worth noting that in all these observed morphologies, the droplets reduce in number from nucleation, growth and coarsening at different spatiotemporal scales. These results support the observation that tuning crowder concentrations gives cells the capability to alter the spatiotemporal self-organization dynamics of MOLs and effectively altering their biophysical properties. To quantify the emergence of these different patterns, we employ the dynamic structure which is the square of modulus of the Fourier transform applied to the field variable $\phi \left(r,t\right)$, that is, $S\left(k,t\right)={\left|\sum_{n}\sum_{m}\left(\phi \left(r_{n,m},t\right)-\langle \phi \rangle \right)\exp\left(-2i\pi \left(nk_{1}-mk_{2}\right)\right)\right|}^{2}$; where $\phi \left(r_{n,m},t\right)$ is the volume fraction at position $r_{m,n}$ at time *t* (*m* and *n* being the lattice node positions), and $k=k_{1}+k_{2}$ is the wave vector in Fourier space [74].

**Figure 7:**
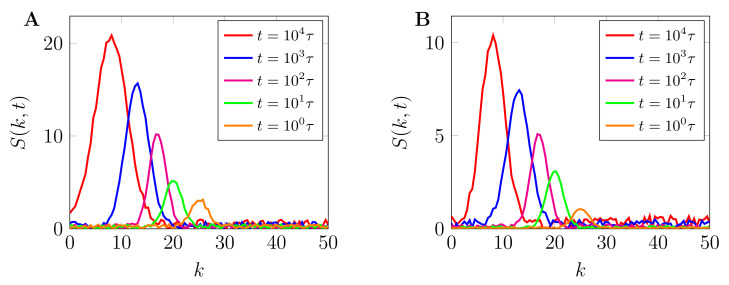
Spherically averaged structure functions. (**A**) Structure function corresponding to Figure 5A and (**B**) structure function corresponding to Figure 6A at different times plotted against the wavenumber $k=\left|k\right|$. As can be observed, both structure functions reveal several controllable characteristic length scales that emerge from the fast and slow coarsening of phase-separated patterns. In both (**A**,**B**), during the early stage of phase separation, the structure function grows exponentially, signifying an almost constant domain size due to the suppressed interconversion of phase separating proteins by macromolecular crowders. The inclusion of chemical reactions reduces the peaks of the structure functions which eventually leads to the arrested coarsening and formation of labyrinthine morphologies, as observed in late stages of Figure 6B,C.

Figure 7 shows the plot of spherically averaged structure factor S(k,t), both with and without chemical reactions (Figure 7A,B, respectively) plotted against the wave number, $k=\left|k\right|$. As can be observed, the structure functions reveal several controllable characteristic length scales that emerge from the fast and slow coarsening of phase-separated patterns. During the early stage of phase separation, both structure function grows exponentially, signifying the quick nucleation and growth of phase-separated morphologies due to the fast mobility in the low macromolecular crowders regime. The inclusion of chemical reactions reduces the peaks of the structure function which eventually leads to the arrested coarsening and formation of labyrinthine morphologies as observed in late stages of Figure 6C. This is further discussed in Section 3.2.

**Figure 8:**
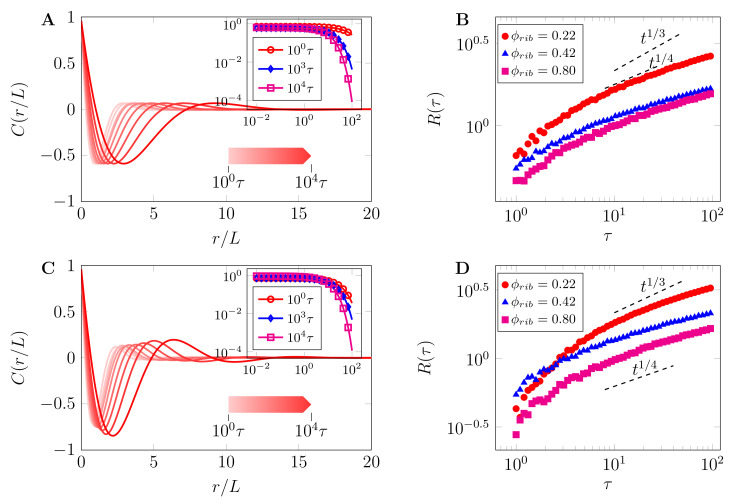
Normalized correlation functions (C(r/L)) and dominant characteristic length-scales ($R\left(t\right)∼At^{\lambda }$): (**A**) correlation function for morphology in Figure 5A, without reactions, that is, R(ϕ)=0 and (**B**) corresponding dominant length-scale for different crowder concentrations. (**C**) Correlation function for morphology in Figure 6A, with reactions, that is, R(ϕ)≠0 and (**D**) corresponding dominant length-scale for different crowder concentrations. As can be noted in both (**B**,**D**), the exponent for early nucleation and growth is predominately higher than that for late stage-coarsening towards steady state regimes, λ∼1/4. For the same value of crowder concentration, the growth and coarsening rates are increased when reactions are included, as can be concluded from both late modes of the correlation functions and late-stage dominant characteristic length-scales. *Insert:* Log–log plots of the same quantities to illustrate scaling for selected times.

To further probe the interplay between the field variable $\phi \left(r,t\right)$ and length-scales of phase-separated morphologies, we compute the correlation function, $C\left(r,t\right)=\langle \phi \left(r,\tau \right)\phi \left(0,\tau \right)\rangle$ where $r=\left|r\right|$, assuming transnational invariance [74]. The correlation function is related to the dominant characteristic length (R(t)) and the structure function by $S\left(k,t\right)=R{\left(t\right)}^{d}\hat{S}\left(kR\left(t\right)\right)$. Figure 8 shows the plot of the normalized correlation functions (C(r/L)) and dominant characteristic length-scales ($R\left(t\right)∼At^{\lambda }$). Figure 8A is the correlation function corresponding to morphology in Figure 5A with R(ϕ)=0 and Figure 8B shows the corresponding dominant length-scale for different crowder concentrations. Similarly, Figure 8C shows the correlation function for morphology in Figure 6A with R(ϕ)≠0 and Figure 8C shows corresponding dominant length-scale for different crowder concentrations. As can be noted from both figures, the exponent for early nucleation and growth is predominately higher than that for late-stage coarsening towards steady state regimes, λ∼1/3. For the same value of crowder concentration, the growth and coarsening rates increase when reactions are included as can be concluded from both late modes of the correlation functions and late-stage dominant characteristic length scales.

### 3.2. Arrest of Domain Coarsening by Chemical Reactions at High Crowder Concentrations

**Figure 9:**
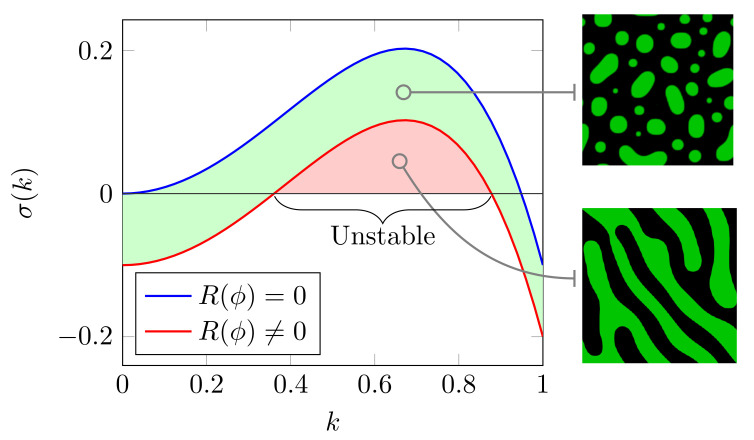
Dispersion characteristics of phase-separated morphologies with and without chemical reactions at late stages of evolution. The inclusion of chemical reactions can result in unstable wave vectors (k∈[0.37,0.87]) that are independent of domain size. These independent states may lead to domain-coarsening arrest, resulting in the phase-separated morphologies changing from droplets to striped patterns akin to anomalous gel-like morphologies observed in experiments.

We now study the effects of chemical reactions on the coarsening of final morphologies at high macromolecular crowding. This was performed by analyzing the dispersion characteristics of the dynamics (13) with and without chemical kinetics. If Equation (13) is linearized around the initial average value of the field variable and Fourier transform, we obtain the growth rate of small perturbations as $\sigma \left(k\right)\approx k^{2}\left(k_{c}^{2}-k^{2}\right)-\frac{1}{k_{0}}R\left(\phi \right)$, where $k_{c}={\left.\partial_{\phi }^{2}f\left(\phi \right)\right|}_{\phi =\phi_{0}}$[67]. As can be observed from Figure 9, the inclusion of chemical reactions can result in unstable wave vectors (k∈[0.37,0.87]) that are independent of domain size. These independent states may lead to domain-coarsening arrest, resulting in the phase-separated morphologies changing from droplets to labyrinthine patterns as shown in Figure 9. Biochemically, the range of parameters *k* may be tuned by both the chemical reactions and the presence of macromolecular crowders. This phase transition is consistent with in vitro experiments where it has been shown that crowder-mediated depletion interactions alter the liquid-like features of condensates to viscoelastic gel-like state [37,59,75].

## 4. Conclusions and Outlook

Macromolecular crowding plays critical roles in regulating in vivo LLPS. For example, the complex rapamycin (mTOR) has been shown to regulate the abundance of ribosomes in the cytoplasm, thereby accelerating or decelerating condensate formation and effectively altering the biophysical properties of the condensed phases [46,47,56]. Although these experiments have shed some light on the effects of macromolecular crowders, the phenomenon still remains poorly understood, primarily because of the complex nature of the intracellular space compared to purified proteins studied in vitro. One way of understanding the roles crowders play in LLPS is to augment experimental and theoretical studies. In this work, we presented a minimal model that couples the conventional Cahn–Hilliard model for phase separation in binary mixtures with crowder concentration-dependent mobility and state-dependent reaction rates. Through extensive field-theoretic computer simulations, we showed that the inclusion of macromolecular crowding results in late-stage coarsening and the stabilization of relatively smaller droplets. We further showed that at a high crowder concentration, the model predicts an accelerated growth and coarsening of droplets. However, any further increase in crowder concentration results in the reactions arresting late-stage coarsening of droplets, thereby effectively forming anomalous labyrinthine morphologies akin to gel-like protein aggregates observed in experiments. These results give a framework on which further theoretical and experimental work can be based on understanding the intricate roles played by LLPS in the self-organization dynamics of cells.

Although the model used herein incorporates crowder concentration and state-dependent chemical reactions, these phenomena have been treated separately and then coupled to phase separating dynamics via the mobility and reaction terms of the Cahn–Hilliard equation. However, macromolecular crowding highly affects chemical reactions [76,77] and therefore future work should look into coupling LLPS by deriving models that connect crowder concentrations and chemical reaction propensities.

## Supplementary Materials

The following are available at https://www.mdpi.com/article/10.3390/ijms22136675/s1: 1. Video S1: Evolution of phase-separated morphology in the absence of chemical reactions and for $\phi_{rib}=0.22$, corresponding to Figure 5A. 2. Video S2: Evolution of phase-separated morphology in the absence of chemical reactions and $\phi_{rib}=0.80$ corresponding to Figure 5C. 3. Video S3: Evolution of phase-separated morphology in the presence of chemical reactions and for $\phi_{rib}=0.22$ corresponding to Figure 6A. 4. Video S4: Evolution of phase-separated morphology in the absence of chemical reactions and for $\phi_{rib}=0.80$ corresponding to Figure 6C.
